# Elevated Cholesterol in the *Coxiella burnetii* Intracellular Niche Is Bacteriolytic

**DOI:** 10.1128/mBio.02313-16

**Published:** 2017-02-28

**Authors:** Minal Mulye, Dhritiman Samanta, Seth Winfree, Robert A. Heinzen, Stacey D. Gilk

**Affiliations:** a Department of Microbiology and Immunology, Indiana University School of Medicine, Indianapolis, Indiana, USA; b Department of Medicine, Indiana Center for Biological Microscopy, Indiana University School of Medicine, Indianapolis, Indiana, USA; c *Coxiella* Pathogenesis Section, Laboratory of Bacteriology, Rocky Mountain Laboratories, National Institute of Allergy and Infectious Diseases, National Institutes of Health, Hamilton, Montana, USA; Harvard Medical School

## Abstract

*Coxiella burnetii* is an intracellular bacterial pathogen and a significant cause of culture-negative endocarditis in the United States. Upon infection, the nascent *Coxiella* phagosome fuses with the host endocytic pathway to form a large lysosome-like vacuole called the parasitophorous vacuole (PV). The PV membrane is rich in sterols, and drugs perturbing host cell cholesterol homeostasis inhibit PV formation and bacterial growth. Using cholesterol supplementation of a cholesterol-free cell model system, we found smaller PVs and reduced *Coxiella* growth as cellular cholesterol concentration increased. Further, we observed in cells with cholesterol a significant number of nonfusogenic PVs that contained degraded bacteria, a phenotype not observed in cholesterol-free cells. Cholesterol had no effect on axenic *Coxiella* cultures, indicating that only intracellular bacteria are sensitive to cholesterol. Live-cell microscopy revealed that both plasma membrane-derived cholesterol and the exogenous cholesterol carrier protein low-density lipoprotein (LDL) traffic to the PV. To test the possibility that increasing PV cholesterol levels affects bacterial survival, infected cells were treated with U18666A, a drug that traps cholesterol in lysosomes and PVs. U18666A treatment led to PVs containing degraded bacteria and a significant loss in bacterial viability. The PV pH was significantly more acidic in cells with cholesterol or cells treated with U18666A, and the vacuolar ATPase inhibitor bafilomycin blocked cholesterol-induced PV acidification and bacterial death. Additionally, treatment of infected HeLa cells with several FDA-approved cholesterol-altering drugs led to a loss of bacterial viability, a phenotype also rescued by bafilomycin. Collectively, these data suggest that increasing PV cholesterol further acidifies the PV, leading to *Coxiella* death.

## IMPORTANCE

The intracellular Gram-negative bacterium *Coxiella burnetii* is a significant cause of culture-negative infectious endocarditis, which can be fatal if untreated. The existing treatment strategy requires prolonged antibiotic treatment, with a 10-year mortality rate of 19% in treated patients. Therefore, new clinical therapies are needed and can be achieved by better understanding *C. burnetii* pathogenesis. Upon infection of host cells, *C. burnetii* grows within a specialized replication niche, the parasitophorous vacuole (PV). Recent data have linked cholesterol to intracellular *C. burnetii* growth and PV formation, leading us to further decipher the role of cholesterol during *C. burnetii*-host interaction. We observed that increasing PV cholesterol concentration leads to increased acidification of the PV and bacterial death. Further, treatment with FDA-approved drugs that alter host cholesterol homeostasis also killed *C. burnetii* through PV acidification. Our findings suggest that targeting host cholesterol metabolism might prove clinically efficacious in controlling *C. burnetii* infection.

## INTRODUCTION

Cholesterol is known to play key roles in cardiovascular disorders, obesity, diabetes, and infectious diseases caused by numerous bacterial, viral, and protozoal pathogens. Intracellular pathogens in particular target cholesterol at various stages of infection. For example, *Mycobacterium bovis* and *Helicobacter pylori* directly target cholesterol as a “docking site” to stabilize interactions with the host cell membrane and initiate internalization (1–3). *Mycobacterium* spp., *Brucella suis*, *Listeria monocytogenes*, *Leishmania donovani*, and *Plasmodium falciparum* appear to target cholesterol-rich lipid rafts during entry into both phagocytic and nonphagocytic cells (3–13). Once inside the cell, cholesterol is often targeted during establishment of the intracellular niche and bacterial growth. For example, *Mycobacterium tuberculosis* and *Mycobacterium leprae* accumulate cholesterol in the early phagosome as a mechanism to inhibit phagosome-lysosome fusion and promote pathogen survival (14–16). *M. tuberculosis* also utilizes a cholesterol import system to hijack host cell cholesterol as a carbon and energy source (17). *Chlamydia trachomatis* intercepts cholesterol trafficking from the Golgi apparatus and incorporates cholesterol into the *Chlamydia*-containing inclusion body as well as the bacterial cell wall (18). Thus, modulation of cellular cholesterol by diverse microbial pathogens appears to play an important role in promoting pathogen entry, survival, and subsequent disease.

Recent reports have implicated cholesterol as an important factor during infection by the intracellular bacterial pathogen *Coxiella burnetii*, a significant cause of culture-negative endocarditis in the United States (19–21). An obligate intracellular pathogen during natural infection, *C. burnetii* forms a unique niche in a modified acidic phagolysosome known as the parasitophorous vacuole (PV). After uptake by the host cell via phagocytosis, the bacterium resides in a tight-fitting nascent phagosome that matures through the default endocytic pathway (22, 23). Approximately 24 to 48 h postinfection, the *C. burnetii* PV expands through fusion with early and late endosomes, lysosomes, and autophagosomes (24). As a result, the mature PV membrane is a hybrid of host vesicular membranes, and the vacuole displays various characteristics of a phagolysosome, including lysosomal hydrolases (acid phosphatase, cathepsin D, and 5′-nucleotidase) and an acidic pH of ~4.5 to 5 (24). Establishment of a replication-competent PV requires the *C. burnetii* Dot/Icm type 4B secretion system (T4BSS), which manipulates host cell trafficking and signaling pathways via the activity of effector proteins secreted into the host cytoplasm (25).

Formation of the *C. burnetii* PV is a highly dynamic process involving vesicular trafficking and fusion events, with the PV membrane playing a central role. A distinguishing feature of the *C. burnetii* PV membrane, based on staining with the fluorescent sterol-binding compound filipin, is that it is rich in sterols (21). A role for cholesterol during *C. burnetii* infection was suggested by gene expression analysis of infected host cells, which found that genes involved in cholesterol efflux and storage are upregulated during *C. burnetii* infection (26, 27). Further, a recent screen of a FDA-approved drug library identified 57 drugs that perturb host cell cholesterol homeostasis also block *C. burnetii* growth in THP-1 human macrophage-like cells (19). Intriguingly, these drugs had a more pronounced effect on *C. burnetii* than on *Legionella pneumophila*, *Rickettsia conorii*, or *Brucella abortus*, suggesting that *C. burnetii* may be uniquely sensitive to altered host cell cholesterol homeostasis. Additionally, when cholesterol transport from endosomes and presumably the *C. burnetii* PV was blocked through knockdown of the cholesterol transporter NPC-1, *C. burnetii* growth was significantly attenuated (19). Together, these studies suggest that cholesterol is an important player affecting the *C. burnetii*-host cell interaction.

In order to further understand the role of cholesterol during *C. burnetii* infection, we developed a novel cholesterol-free host cell tissue culture system using cells lacking DHCR24, the final enzyme in cholesterol biosynthesis (20). When adapted to serum-free media, DHCR24^−/−^ mouse embryonic fibroblasts lack both endogenous and exogenous cholesterol sources, and instead, they accumulate desmosterol in cellular membranes. Cholesterol-free cells are an attractive model for deciphering the role of cholesterol in cellular processes, enabling cholesterol manipulation by the addition of exogenous cholesterol to the media. Our prior studies with this model system revealed that *C. burnetii* uptake into fibroblast cells was dependent on cholesterol-rich lipid rafts and the integrin α_v_β_3_ (20). Strikingly, *C. burnetii* PV formation and intracellular replication did not require cholesterol. Further, the PV acquired the typical PV markers Rab7, flotillin-2, syntaxin 7, syntaxin 8, and Vamp7 and contained active cathepsin, indicating that the majority of PV maturation events occurred in the absence of cholesterol. However, the lack of the late endosomal marker CD63 in the PV lumen in cholesterol-free cells suggests that cholesterol regulates one or more intracellular trafficking pathways to the PV (20).

While studies thus far indicate that cholesterol plays a key role during *C. burnetii* infection, how cholesterol affects the formation and maintenance of the PV, as well as *C. burnetii* growth, is not yet known. Here, we utilized cholesterol-free cells to further decipher the role of cholesterol in *C. burnetii*-host cell interactions. Our studies surprisingly revealed that increasing cholesterol in the *C. burnetii* PV inhibits fusion between the PV and endosomes, acidifies the PV, and results in *C. burnetii* degradation. Our data demonstrating a cholesterol-mediated negative effect on an intracellular bacterial pathogen is novel and may have broader implications in the treatment of *C. burnetii* infection.

## RESULTS

### PV size and *C. burnetii* growth are sensitive to cholesterol.

DHCR24^−/−^ mouse embryonic fibroblast cells (MEFs) lack the final enzyme in cholesterol biosynthesis. When adapted to serum-free media, these cells are cholesterol free and accumulate desmosterol in place of cholesterol (20). Cellular cholesterol levels can be controlled by adding cholesterol to the culture medium, which is then preferentially incorporated into cellular membranes over desmosterol (20). Using this model system, we can observe the effect of cellular cholesterol on *C. burnetii*-host interactions over a range of cholesterol concentrations and longer infection periods than those used in traditional methods of manipulating cellular cholesterol.

**FIG 1:**
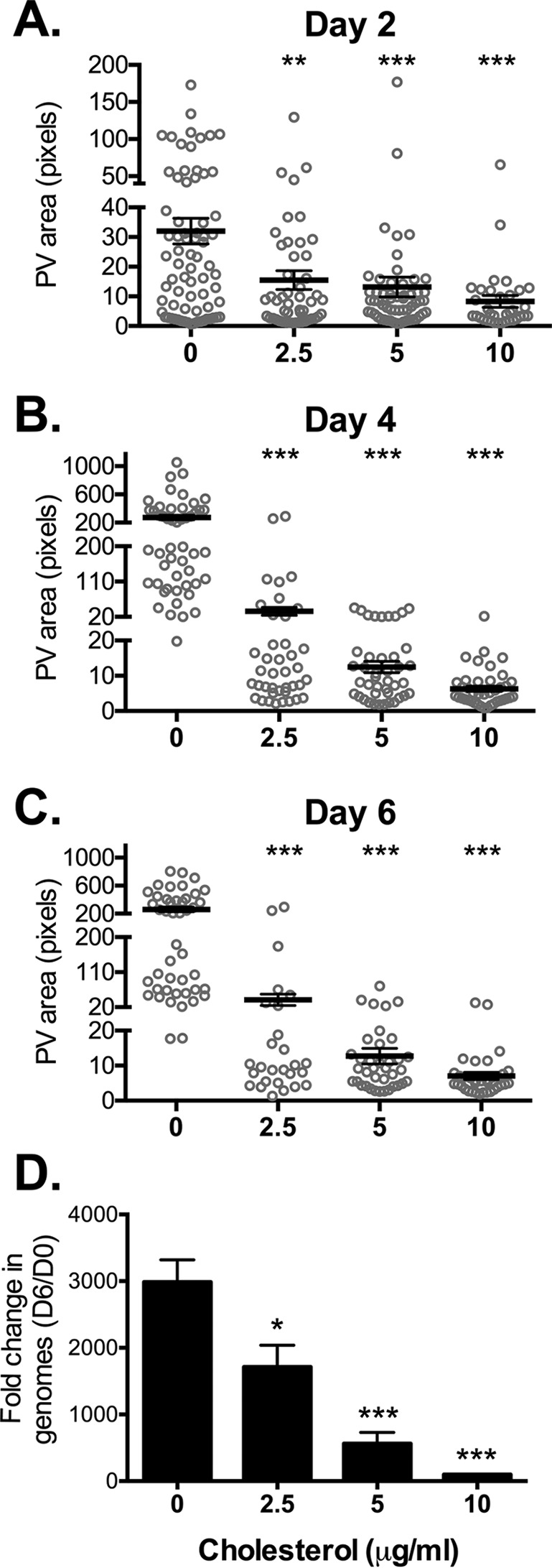
PV size and *C. burnetii* growth are sensitive to cholesterol. (A to C) Measurement of PV sizes reveals that PVs are significantly smaller in MEFs with cholesterol compared to cholesterol-free MEFs. *C. burnetii*-infected MEFs were incubated with the different cholesterol concentrations (0, 2.5, 5, or 10 µg/ml) and stained by immunofluorescence for *C. burnetii* and the PV marker LAMP-1 at the indicated times (2, 4, and 6 days). PVs were measured using ImageJ. Each circle represents the value for 1 PV, with at least 15 PVs per condition measured in each of three separate experiments. The means (black horizontal bars) were compared by one-way ANOVA with Tukey’s posthoc test. Error bars represent the standard errors of the means (SEM). The values that were significantly different from the control values (no cholesterol) are indicated by asterisks as follows: **, *P* < 0.01; ***, *P* < 0.001. (D) The fold change in bacterial growth under different cholesterol conditions was determined by quantitative PCR for bacterial genomes. The means plus standard deviations (SD) from three separate experiments done in duplicate are shown. Values that are significantly different from the control value (no cholesterol) determined by one-way ANOVA with Dunnett’s posthoc test are indicated by asterisks as follows: *, *P* < 0.05, ***, *P* < 0.001. D6, day 6; D0, day 0.

We previously found that while *C. burnetii* entry was reduced, bacterial replication did not appear to be significantly affected in the absence of cholesterol (20). To determine whether host cholesterol levels influence formation of the *C. burnetii* PV, we infected DHCR24^−/−^ MEFs grown under different cholesterol concentrations and assessed PV size over 6 days of infection using immunofluorescence microscopy. Infected cells were fixed and stained using antibodies against *C. burnetii* and LAMP-1 (lysosome-associated membrane glycoprotein 1), a lysosomal protein found on the PV membrane. Surprisingly, at the beginning of PV expansion at 2 days postinfection, the average PV size in cholesterol-free MEFs was at least twice as large as PVs in MEFs with cholesterol (Fig. 1A). While PVs in cholesterol-free MEFs continued to expand approximately 8-fold over the next 4 days, PVs in MEFs with cholesterol remained significantly smaller regardless of the cholesterol concentration (Fig. 1B and C). Given the dramatic effect of cholesterol on PV size, we used quantitative PCR to measure *C. burnetii* growth. The fold change in *C. burnetii* growth over 6 days decreased in a cholesterol-dependent manner, with little growth seen at the highest cholesterol concentration (Fig. 1D). Together, these data indicate that *C. burnetii* PV size and bacterial growth are negatively affected by cholesterol.

### Addition of cholesterol leads to *C. burnetii* lysis.

**FIG 2:**
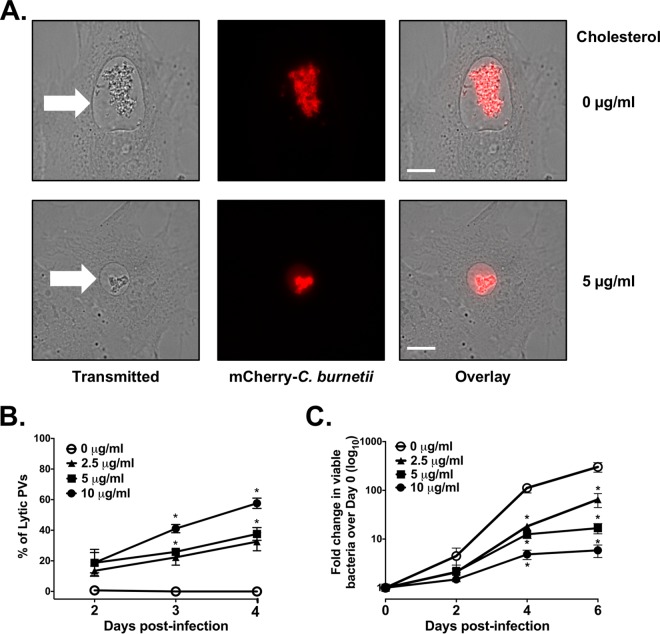
Increasing cellular cholesterol leads to *C. burnetii* death**.** (A) Representative live-cell microscopy images of cholesterol-free MEFs and MEFs with cholesterol and infected with mCherry-expressing *C. burnetii* (mCherry-*C. burnetii*). Note the presence of mCherry fluorescence in the PV lumen in MEFs with cholesterol. The white arrows point to the PVs. Bars = 10 µm. (B) Quantitation of lytic PVs containing degraded bacteria under different cholesterol conditions. At different times postinfection, PVs were observed by live-cell microscopy and scored as lytic if visible mCherry fluorescence was present in the lumen. The means ± SEM from three experiments are shown. The means were compared by one-way ANOVA with Tukey’s posthoc test. *, *P* < 0.05 compared to the value with no cholesterol. (C) Cholesterol leads to fewer viable bacteria. *C. burnetii*-infected cholesterol-free MEFs were grown with different cholesterol concentrations, and the number of viable bacteria was determined by fluorescent infectious focus-forming unit (FFU) assay. Error bars show the SEM of the averages of three individual experiments done in duplicate. Means were compared by one-way ANOVA with Tukey’s posthoc test. *, *P* < 0.05 compared to the value with no cholesterol.

Fixed-cell microscopy suggested that poor bacterial growth in the presence of cholesterol was associated with deficient PV formation, similar to the observed phenotype for *C. burnetii* T4BSS mutants (28, 29). To further examine this phenotype, we used live-cell imaging of MEFs with or without cholesterol and infected with mCherry-expressing *C. burnetii* (mCherry-*C. burnetii*). Surprisingly, we observed in MEFs with cholesterol a significant number of PVs with free mCherry fluorescence in the PV lumen, a result of lysis or degradation of mCherry-expressing *C. burnetii* (Fig. 2A, bottom panel). Because the bacteria have lysed, we have designated these PVs “lytic.” By 2 days postinfection, approximately 20% of the PVs were lytic in MEFs with cholesterol, regardless of the cholesterol concentration. Over the next 48 h, the percentage of lytic PVs in MEFs with cholesterol increased in a dose-dependent fashion. Importantly, we never observed lytic PVs in cholesterol-free MEFs (Fig. 2B). To determine whether live-cell microscopy data correlated with bacterial viability, we measured viable bacteria using a fluorescent infectious focus-forming unit (FFU) assay (30). Bacteria were recovered from MEFs under different cholesterol conditions, replated onto a monolayer of Vero cells, and incubated for 5 days. After *C. burnetii* bacteria were stained, the numbers of fluorescent foci were counted, with one focus unit equivalent to one viable bacteria. Over a period of 6 days, the number of viable *C. burnetii* in MEFs with cholesterol decreased 80 to 98% compared to the number in cholesterol-free MEFs, depending on the cholesterol concentration (Fig. 2C). These data suggest that, as opposed to a lack of bacterial growth, the addition of cholesterol to cholesterol-free MEFs is bacteriolytic.

### Cholesterol traffics to the *C. burnetii* PV.

We next examined cholesterol trafficking in cholesterol-free MEFs to determine how the addition of exogenous cholesterol in our model system might lead to *C. burnetii* death. Exogenous cholesterol could be internalized into the cell through two mechanisms: insertion into the plasma membrane or uptake of cholesterol-bound low-density lipoprotein (LDL). In the first case, cholesterol intercalates into the plasma membrane and is then distributed throughout the cell via vesicular and nonvesicular pathways. This pathway also mimics trafficking of endogenous host cell cholesterol, which is synthesized in the endoplasmic reticulum (ER) and then transported to the plasma membrane for cellular distribution. To examine whether plasma membrane-derived cholesterol travels to the PV, we incubated *C. burnetii*-infected DHCR24^−/−^ MEFs with fluorescent BODIPY-cholesterol complexed to methyl-β-cyclodextrin, which leads to incorporation of the BODIPY-cholesterol into the plasma membrane. After 24 h incubation at 37°C to allow for cellular trafficking, live-cell microscopy revealed fluorescent cholesterol in vesicular structures and the *C. burnetii* PV (see Fig. S1A in the supplemental material). We next examined trafficking of exogenous LDL, a major cholesterol-binding protein internalized by cells through receptor-mediated endocytosis. Following incubation of infected DHCR24^−/−^ MEFs with fluorescent LDL for 4 h, fluorescent LDL was found in the PV membrane and PV lumen (Fig. S1B). Thus, at least two sources of cholesterol travel to the PV, suggesting that cholesterol supplementation of cholesterol-free MEFs increases PV cholesterol.

10.1128/mBio.02313-16.2 FIG S1  Cholesterol traffics to the *C. burnetii* PV. (A and B) Live-cell microscopy images showing that plasma membrane-derived BODIPY-cholesterol (A) and BODIPY-LDL (B) traffics to the *C. burnetii* PV (boxed insets) in MEFs. Cholesterol or LDL is indicated in green, and mCherry-*C. burnetii* is shown in red. The arrows point to PVs. Download FIG S1, TIF file, 11.8 MB. Copyright © 2017 Mulye et al. 2017 Mulye et al. This content is distributed under the terms of the Creative Commons Attribution 4.0 International license.

### U18666A-induced cholesterol accumulation in the PV is bacteriolytic.

**FIG 3:**
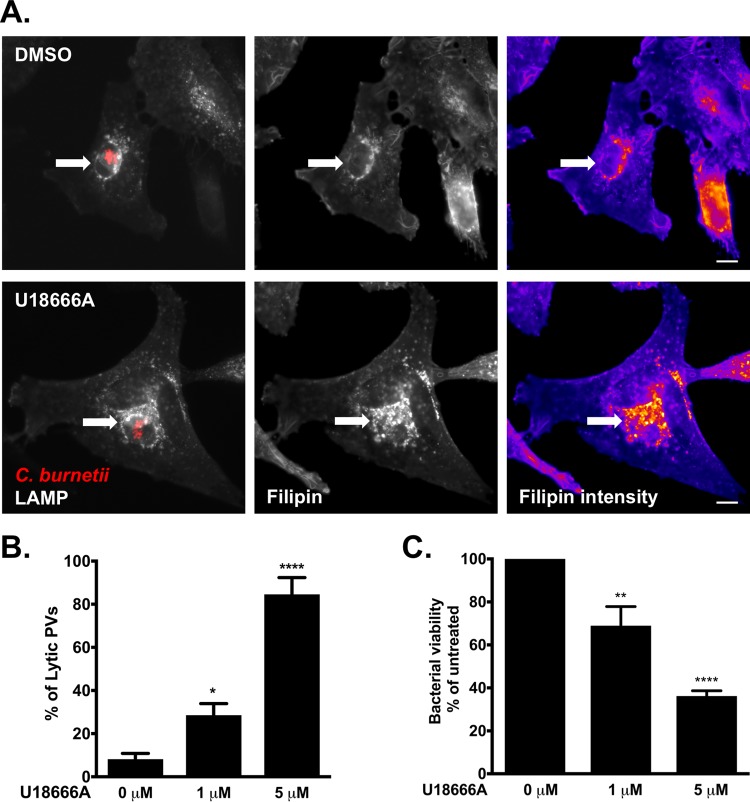
Altered cellular cholesterol homeostasis is bactericidal. (A) Microscopy images showing that U18666A treatment traps cholesterol in the *C. burnetii* PV in HeLa cells. mCherry-*C. burnetii*-infected HeLa cells were treated with 5 µM U18666A for 6 h, fixed, and stained for sterols (filipin) and PV (LAMP-1). Compared to mock-treated cells, there is an increase in filipin labeling in and around the PV following treatment with U18666A. The white arrows point to the PVs. Filipin intensity is shown as a heat map, with yellow showing the highest filipin intensity and blue showing the lowest filipin intensity. Bars = 5 µm. (B) Quantitation of lytic PVs in U18666A-treated cells after treatment of mCherry-*C. burnetii-*infected HeLa cells with 1 or 5 µM U18666A. PVs were scored for the presence (lytic) or absence (nonlytic) of free mCherry in the PV lumen, resulting from the lysis of mCherry-expressing bacteria. The means plus SEM (error bars) from three individual experiments are shown. The means were compared to the value with no cholesterol by one-way ANOVA with Dunnett’s posthoc test, and statistically different values are indicated by asterisks as follows: *, *P* < 0.05; ****, *P* < 0.0001. (C) *C. burnetii* viability decreases after 6 h treatment with U18666A. The number of viable bacteria was determined by FFU assay and normalized to the values for the vehicle control (0 µM). The means plus SEM (error bars) from three individual experiments are shown. Statistical significance was determined by comparing values to the value with no cholesterol by one-way ANOVA with Dunnett’s posthoc test and indicated as follows: **, *P* < 0.01; ****, *P* < 0.0001. The average values for three independent experiments done in duplicate are shown.

On the basis of our cholesterol trafficking data, we hypothesized that accumulation of cholesterol in the PV leads to *C. burnetii* death. The cholesterol-altering drug U18666A blocks cholesterol transport from endosomes and lysosomes and results in cholesterol accumulation in the endolysosomal system (31). To determine whether U18666A also leads to cholesterol accumulation in the PV, we treated infected HeLa cells with U18666A and then stained with filipin to label cholesterol and antibodies against LAMP-1 and *C. burnetii*. As previously shown, filipin staining of untreated infected cells shows the presence of cholesterol or other sterols in the PV membrane (Fig. 3A, top). However, after U18666A treatment, there is a significant increase in filipin labeling in and around the PV (Fig. 3A, bottom), suggesting an increase in PV cholesterol. By live-cell microscopy, we observed a significant number of lytic PVs shortly after adding U18666A to mCherry-*C. burnetii*-infected HeLa cells. When quantitated after a 6 h treatment of either 1 µM and 5 µM U18666A, approximately 30% and 85% of PVs were lytic, respectively (Fig. 3B). Furthermore, the presence of lytic PVs corresponded with a 30 to 60% loss of bacterial viability, depending on the U18666A concentration (Fig. 3C). Cumulatively, these data suggest that U18666A-induced cholesterol accumulation in the PV leads to *C. burnetii* death.

To address the possibility that cholesterol could be directly killing *C. burnetii*, we measured growth of axenic *C. burnetii* cultures in the presence or absence of cholesterol. Compared to the carrier protein bovine serum albumin (BSA), cholesterol had no effect on *C. burnetii* growth (Fig. S2A). Further, the bacteriolytic effect of U18666A is not due to a direct effect on *C. burnetii*, as the addition of U18666A to *C. burnetii* axenic cultures did not affect viability (Fig. S2B). These data indicate that the toxic effect of cholesterol and U18666A is specific to the *C. burnetii* intracellular niche.

10.1128/mBio.02313-16.3 FIG S2  The bactericidal effect of cholesterol and U18666A is specific to intracellular bacteria. (A) Cholesterol addition to axenic *C. burnetii* cultures does not affect bacterial growth. BSA-cholesterol or BSA alone was added to cultures, and the number of bacterial genomes was determined using quantitative PCR every day for a 4 day culture. The results of a representative experiment, done in duplicate, are shown. The means ± SD (error bars) are shown. (B) U18666A does not directly kill *C. burnetii*. Axenic *C. burnetii* cultures grown for 3 days were treated for 6 h with U18666A, and bacterial viability was determined using a FFU assay and normalized to vehicle (0 µM) cultures. The means ± SEM (error bars) from three experiments performed in duplicate are shown. ns, not significant compared to the value for the vehicle control as determined by one-way ANOVA with Tukey’s posthoc test. Download FIG S2, TIF file, 10.4 MB. Copyright © 2017 Mulye et al. 2017 Mulye et al. This content is distributed under the terms of the Creative Commons Attribution 4.0 International license.

### *C. burnetii* growth is most sensitive to cholesterol during the early stages of PV biogenesis.

**FIG 4:**
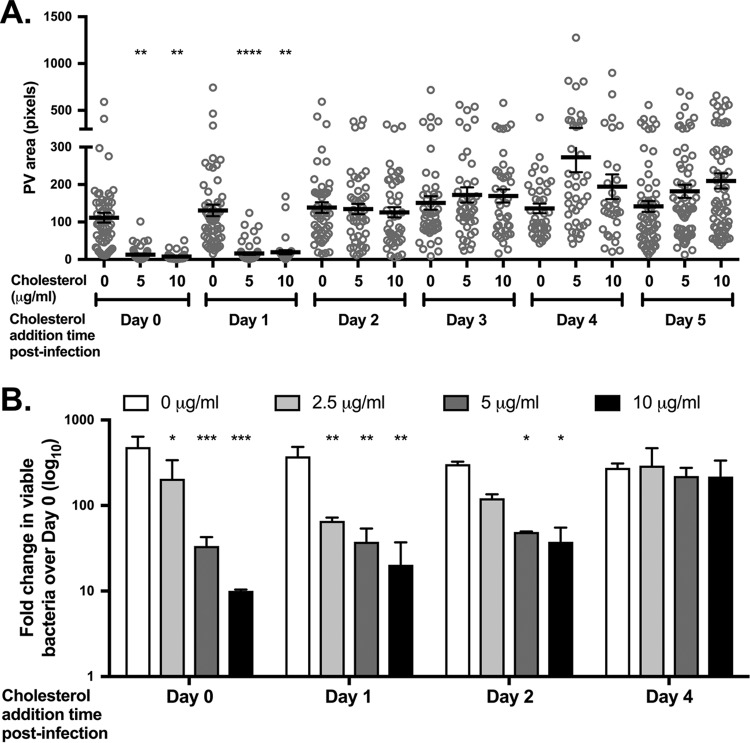
*C. burnetii* growth is sensitive to cholesterol during early stages of PV biogenesis. (A) Final PV size after adding cholesterol at various times postinfection in MEFs. Cholesterol-free MEFs were infected with *C. burnetii* and different cholesterol concentrations were added to the cells each day from day 0 to 5. At day 6, cells were fixed and stained for the PV marker LAMP-1 and *C. burnetii*, and PV size was measured using ImageJ. At least 20 PVs were measured for each condition for three independent experiments. Each circle indicates the value for an individual PV. The means (black bars) ± SEM (error bars) from three individual experiments are shown. Statistical significance was determined by comparing values to the value with no cholesterol by one-way ANOVA with Dunnett’s posthoc test and indicated as follows: **, *P* < 0.01; ****, *P* < 0.0001. (B) Recoverable bacteria at day 6 post infection after cholesterol addition at various times postinfection in MEFs. *C. burnetii*-infected cholesterol-free MEFs were grown with different cholesterol concentrations added at different times postinfection. Bacterial viability was measured at day 6 by FFU assay. The results shown are representative of three separate experiments performed in duplicate. The means plus SD (error bars) are shown. Statistical significance was determined by comparing values to the value with no cholesterol by two-way ANOVA with Tukey’s posthoc test and indicated as follows: *, *P* < 0.05; **, *P* > 0.001; ***, *P* < 0.001.

During the first 24 to 48 h of infection, *C. burnetii* resides in a tight-fitting PV, as the nonreplicating small-cell variant (SCV) transitions into the replication-competent large-cell variant (LCV). During this time, the *C. burnetii* T4SS also secretes effector proteins into the host cell cytoplasm, with secretion detected as early as 8 h postinfection in HeLa cells and 1 h postinfection in mouse bone marrow-derived macrophages (32). PV expansion around 48 h postinfection coincides with the beginning of log-phase growth; LCVs transition back to SCVs around 5 or 6 days postinfection (33). To determine whether *C. burnetii* is sensitive to cholesterol at specific stages of infection, we added cholesterol to MEFs at 24 h intervals postinfection and assessed the effect on PV size and bacterial viability. When cholesterol was added at the time of infection or 1 day postinfection, the final PVs at 6 days postinfection were significantly smaller than those in cholesterol-free MEFs (Fig. 4A). However, there was no significant effect on final PV size when cholesterol was added at any time after 1 day postinfection, regardless of the cholesterol concentration. Similarly, the fold change in recoverable bacteria over 6 days was sensitive to cholesterol only during the first 2 days of infection, with an approximately 80% decrease in bacterial viability in MEFs with cholesterol compared to cholesterol-free MEFs (Fig. 4B). While not statistically significant, cholesterol addition after day 4 trended toward slightly larger PVs, although there was no effect on bacterial viability. Based on these data, *C. burnetii* is sensitive to cholesterol only during the early stages of PV expansion and log growth.

### Lytic PVs are nonfusogenic.

**FIG 5:**
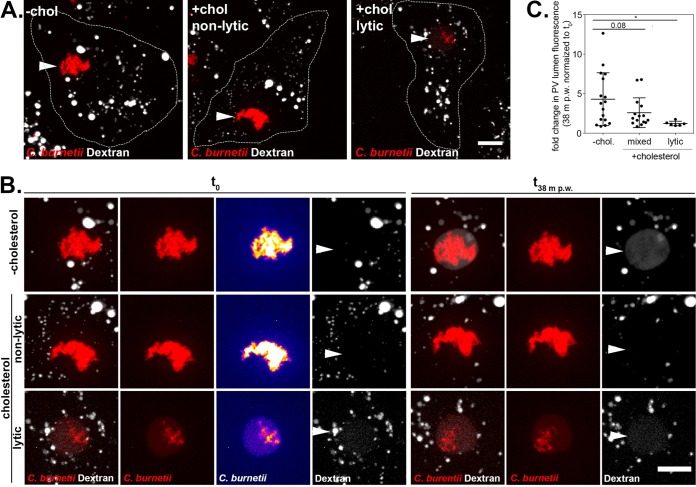
Lytic PVs are nonfusogenic. (A and B) Representative microscopy images showing increased dextran accumulation in the lumen of PVs from MEFs grown in cholesterol-free media (−chol) compared to cells grown in media with cholesterol (+chol). The cells are outlined in white in panel A, while panel B shows 2.5× insets of the PV and surrounding cytoplasm from panel A. MEFs infected with mCherry-expressing *C. burnetii* were pulsed with fluorescent dextran for 10 min, washed with media to remove noninternalized dextran, followed by imaging of PVs every 6.3 min for 38 min. *C. burnetii* bacteria are shown in red, and dextran is shown in white. The maximum *z*-axis projections are shown. Bars = 10 µm. In the *t*0 *C*. *burnetii* column in panel B, the *C. burnetii* image was mapped to an intensity lookup table to visualize the lytic phenotype, with blue depicting the lowest fluorescent intensity and yellow showing the highest intensity. Note the presence of red fluorescence in the PV lumen of the lytic PV. PVs are indicated by white arrowheads. The images were processed identically, and the gamma was increased to 1.5 to more easily visualize PV dextran. (C) The fold change in total PV lumen dextran fluorescence intensity at the end of the time course versus time zero (*t*0) for the volume at 38 min postwashing (38 m p.w.). Each circle indicates the value for an individual PV, and the means ± standard deviations of the means for groups of PVs are shown. Statistical significance was determined by an unpaired *t* test and indicated as follows: *, *P* < 0.05.

The *C. burnetii* PV is a highly dynamic vacuole, promiscuously fusing with vesicles from the endocytic pathway (34). We previously observed cholesterol-dependent fusion between late endosomes and the PV, based on a lack of the late endosome marker CD63 in the PV lumen in cholesterol-free MEFs (20). To further characterize the effect of cholesterol on endosomal trafficking to the PV, we developed a quantitative fusogenicity assay utilizing fluorescent dextran. Dextran is internalized by cells through non-receptor-mediated endocytosis and accumulates in the PV lumen following fusion between endosomes and the PV (35). To measure PV-endosome fusion, we quantitated dextran accumulation in the PV lumen in MEFs with or without cholesterol. MEFs infected with mCherry-*C. burnetii* were pulsed for 10 min with fluorescent dextran and then imaged for 40 min using live-cell confocal microscopy (Fig. 5A and B). The accumulation of dextran in the PV lumen in individual PVs was determined by measuring the fold change in fluorescence intensity over 40 min. An average of 4.2-fold increase in fluorescent dextran was observed in PVs from cholesterol-free MEFs (Fig. 5C). In cells with cholesterol, PVs that were not yet lytic were less fusogenic, with an average of 2.5-fold increase in fluorescent dextran. In contrast, lytic PVs (defined as having a twofold increase in PV lumen mCherry fluorescence compared to background) accumulated little to no fluorescent dextran, indicating that PVs containing degraded bacteria are no longer fusogenic with the endocytic pathway.

### Cholesterol further acidifies the *C. burnetii* PV.

**FIG 6:**
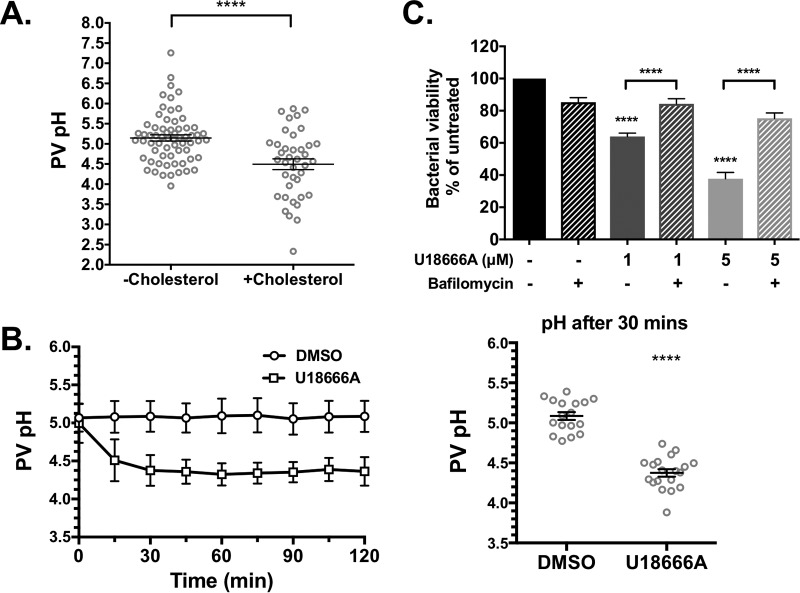
Cholesterol accumulation in the PV increases PV acidity. PV pH was determined at 3 days postinfection using a ratiometric fluorescence assay of pH-sensitive Oregon Green dextran and pH-insensitive Alexa 647 dextran. (A) The pH in PVs from MEFs with cholesterol (average pH 4.5) was significantly more acidic than PVs from cholesterol-free MEFs (average pH 5.2). At least 10 PVs were measured in three separate experiments. The means ± SEM (error bars) from three individual experiments are shown. ****, *P* < 0.0001 as determined by two-tailed unpaired *t* test. (B) Average PV pH over 2 h in HeLa cells treated with DMSO or 5 µM U18666A. PVs were identified by microscopy and imaged prior to adding drug. While PV pH in DMSO-treated cells remained stable over the time course, PVs in U18666A-treated cells further acidified in the first 30 min. The average pH values at 30 min were 5.1 in DMSO-treated cells and 4.4 in U1666A-treated cells. Approximately 10 PVs were measured in each of two separate experiments, and individual traces are shown in Fig. S4 in the supplemental material. The averages ± SD (error bars) are shown. All time points were significant (*P* < 0.0001). (C) *C. burnetii*-infected HeLa cells were treated with U18666A (1 µM or 5 µM) and/or the vATPase inhibitor bafilomycin A1 (100 nM) for 3 h. Bacterial viability, as measured by the FFU assay, is rescued in the presence of bafilomycin. The means plus SEM (error bars) from three individual experiments are shown. ****, *P* < 0.0001 for the value for U18666A treatment alone compared to the value for no treatment as determined by one-way ANOVA with Tukey’s posthoc test.

Experiments in cholesterol-rich macrophages have shown that cholesterol affects the ability of lysosomes to maintain an acidic pH (36), raising the possibility that cholesterol influences PV pH. Prior studies using ratiometric pH measurements with fluorescein found the *C. burnetii* PV pH to be approximately 4.8 (37, 38). Further, *C. burnetii* metabolism is activated by acidic pH (39, 40), and blocking PV acidification during phagosome maturation using the vacuolar ATPase (vATPase) inhibitor bafilomycin A1 inhibits bacterial growth (35). Collectively, these data demonstrate that PV pH is a critical component of *C. burnetii* pathogenesis. To determine whether PV pH is affected by cholesterol, we measured PV pH under different cholesterol conditions using a microscopy-based ratiometric fluorescence assay (41). The pH-sensitive fluorophore Oregon Green 488 is a fluorescein derivative with a pK_a_ of 4.7, making Oregon Green 488 more accurate in acidic environments compared to fluorescein (pK_a _of 6.4) (41). MEFs with and without cholesterol were infected with mCherry-*C. burnetii* for 3 days and then incubated with pH-sensitive Oregon Green 488 dextran and pH-insensitive Alexa Fluor 647 dextran for 4 h to allow for dextran trafficking to the PV. It is important to note that because this assay relies on dextran trafficking to the PV, only nonlytic PVs in MEFs with cholesterol can be analyzed, as lytic PVs are nonfusogenic (Fig. 5). The fluorescence intensities of Oregon Green 488 and Alexa Fluor 647 were measured for each PV, and a ratio of Oregon Green 488 to Alexa Fluor 647 was compared to a standard curve to generate individual PV pH measurements. In cholesterol-free MEFs, the average PV pH was 5.1 with a range of 3.95 to 7.26 (Fig. 6A and Fig. S3). Interestingly, PVs in MEFs with cholesterol were significantly more acidic, ranging between 2.33 and 6.02 and an average pH of 4.4.

10.1128/mBio.02313-16.4 FIG S3  Representative images for pH measurements of U18666A-treated PVs. (A and B) Representative images of Oregon Green (OG) fluorescence in DMSO-treated (A) and U18666A-treated (B) HeLa cells infected with *Coxiella burnetii*. At 30 min after the drug was added, the Oregon Green fluorescence quenches in U18666A cells in response to PV acidification. In contrast, Oregon Green fluorescence does not change in DMSO-treated cells. Alexa Fluor 647 is not sensitive to pH. Download FIG S3, TIF file, 21.1 MB. Copyright © 2017 Mulye et al. 2017 Mulye et al. This content is distributed under the terms of the Creative Commons Attribution 4.0 International license.

Given that U18666A also leads to lytic PVs, we measured PV pH before and after the addition of U18666A to infected HeLa cells. After incubation with Oregon Green 488 dextran and Alexa Fluor 647 dextran for 4 h, PVs were imaged prior to adding dimethyl sulfoxide (DMSO) or U18666A and then imaged every 15 min for 2 h. Similar to cholesterol-free MEFs, the average PV pH prior to drug addition was approximately 5.1 (Fig. 6B and Fig. S4). While DMSO-treated cells maintained this pH over 2 h, in cells treated with U18666A, the PV further acidified to an average pH of 4.4 within 30 min.

10.1128/mBio.02313-16.5 FIG S4  pH measurements of individual U18666A-treated PVs. The change in the pH of individual PVs following treatment with DMSO or U18666A is shown. Download FIG S4, TIF file, 9.5 MB. Copyright © 2017 Mulye et al. 2017 Mulye et al. This content is distributed under the terms of the Creative Commons Attribution 4.0 International license.

While these data suggest that cholesterol accumulation leads to PV acidification, we next tested whether increased acidification was responsible for *C. burnetii* degradation. The proton pump vacuolar ATPase is responsible for PV acidification and can be blocked using bafilomycin A1 (35). Bafilomycin A1 recovered bacterial viability in the presence of U18666A (Fig. 6C), indicating that increased acidification of the PV leads to *C. burnetii* death.

### Cholesterol-altering FDA-approved drugs lead to *C. burnetii* lysis.

**FIG 7:**
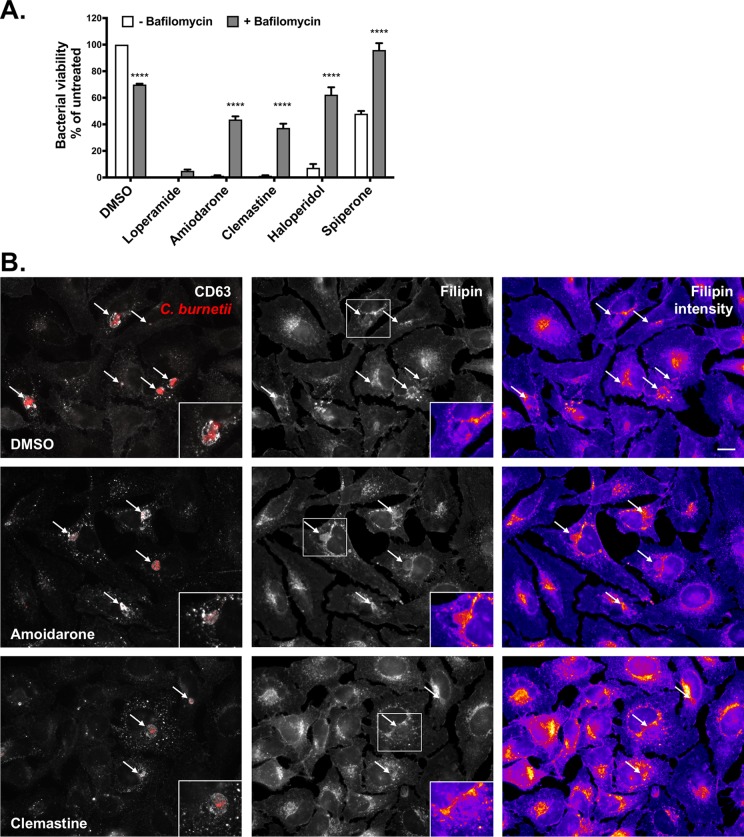
Cholesterol-altering FDA-approved drugs kill *C. burnetii*. *C. burnetii*-infected HeLa cells (3 days postinfection) were treated for 3 h with the indicated drug (20 µM) with or without the vATPase inhibitor bafilomycin A1 (100 nM). (A) Blocking acidification with bafilomycin at least partially rescues bacterial killing by amiodarone, clemastine, haloperidol, and spiperone. Values were normalized to the value with DMSO and without bafilomycin, and the means plus SEM from three experiments are shown. ****, *P* < 0.00001 compared to the value without bafilomycin, as determined by two-way ANOVA with Sidek’s multiple-comparison test. (B) Filipin labeling of mCherry-*C. burnetii*-infected HeLa cells suggests that amiodarone and clemastine alter cholesterol trafficking. In amiodarone-treated cells, more filipin labeling in PVs is observed, while clemastine-treated cells show an overall increase in filipin labeling around the PV. Coverslips were fixed following drug treatment for 3 h, and the cells were stained with filipin (cholesterol) and CD63. Filipin images were taken under identical capture settings and processed identically in ImageJ. The filipin fluorescence intensity is shown using a lookup table, with blue showing the lowest fluorescence intensity and yellow showing the highest fluorescence intensity. The white arrows point to PVs, and representative PVs are shown in the insets. Bars = 10 µm.

A previous study discovered that numerous drugs from a FDA-approved drug library altered cholesterol homeostasis in HeLa cells and inhibited intracellular *C. burnetii* growth (19). To determine whether these drugs worked through a mechanism similar to that of U18666A, we tested a small subset of drugs for their ability to induce lytic PVs in HeLa cells. These drugs were chosen based on their ability to (i) cause cholesterol accumulation in endosomes, (ii) block intracellular *C. burnetii* growth, (iii) not affect *C. burnetii* growth in axenic media, and (iv) have low toxicity to host cells (19) (Table S1). Six of the eight drugs we tested led to lytic PVs after a 3 h treatment (Fig. S5A). In particular, loperamide, clemastine, and amiodarone resulted in nearly all of the PVs containing degraded bacteria, with few intact bacteria (Fig. S5B and data not shown). These observations correlated with almost no bacteria recovered after 3 h, as measured by a FFU assay (Fig. 7A). Furthermore, concurrent incubation with bafilomycin A1 at least partially rescued killing by amiodarone, clemastine, haloperidol, and spiperone, as measured by a decrease in the number of lytic PVs (Fig. S5A) and an increase in bacterial viability (Fig. 7A). This suggests that similar to U18666A, these drugs increase the acidity of the PV.

10.1128/mBio.02313-16.1 TABLE S1  FDA-approved cholesterol-altering drugs. The effects of FDA-approved drugs on *C. burnetii* intracellular and axenic growth and endogenous cholesterol accumulation in treated cells (19) are shown. Download TABLE S1, DOCX file, 0.1 MB. Copyright © 2017 Mulye et al. 2017 Mulye et al. This content is distributed under the terms of the Creative Commons Attribution 4.0 International license.

10.1128/mBio.02313-16.6 FIG S5  Lytic PVs and filipin labeling after treatment with cholesterol-altering FDA-approved drugs. (A) Quantitation of lytic PVs following drug treatment (20 µM) with or without bafilomycin A1 (100 nM). Blocking acidification with vATPase at least partially rescued the presence of lytic PVs for butaclamol, haloperidol, loperamide, clemastine, and amiodarone. The means ± SEM from two experiments are shown. (B) Filipin labeling of infected HeLa cells following 3 h drug treatment. The arrows point to PVs. Bar, 10 µm. Download FIG S5, TIF file, 25.2 MB. Copyright © 2017 Mulye et al. 2017 Mulye et al. This content is distributed under the terms of the Creative Commons Attribution 4.0 International license.

To determine whether these drugs led to altered cholesterol levels in the PV, we observed cholesterol by staining with filipin. While all of the drugs tested appeared to alter cholesterol distribution, several phenotypes were observed (summarized in Table S1). Compared to DMSO-treated cells, amiodarone did not significantly alter overall fillipin labeling intensity of endosomes. However, unlike filipin labeling being restricted to the PV membrane of control cells, amiodarone treatment led to significant filipin labeling of the PV lumen (Fig. 7B, insets). In contrast, filipin primarily labeled the PV membrane in clemastine-treated cells, but there was a significant increase in endosomal filipin labeling intensity. Haloperidol-treated cells had a phenotype similar to that of clemastine-treated cells, while the phenotype of loperamide-treated cells was more similar to that of U18666A-treated cells (Fig. S5). Intriguingly, spiperone-treated cells appeared to have filipin levels similar to those in control cells, with some of the PVs showing labeling within the PV lumen (Fig. S5). Together with the bafilomycin data, this suggests that there might be multiple mechanisms by which altering host cholesterol leads to increased acidification of the *C. burnetii* PV, and ultimately, death of the bacteria. Interestingly, bafilomycin did not rescue the effects of loperamide, suggesting that this drug may act through a different mechanism.

## DISCUSSION

Cholesterol is a critical lipid constituent of cellular membranes, regulating membrane dynamics, trafficking, and signaling. Due to its involvement in important host cell processes, an increasing number of pathogens, including *Leishmania* spp., *Salmonella enterica*, *Staphylococcus aureus*, *Mycobacterium* spp., and *Listeria monocytogenes*, have been reported to exploit host cell cholesterol (3–5, 11–13). To understand the role of cholesterol during *C. burnetii*-host interaction, we utilized a cholesterol-free tissue culture model system that lacks both endogenous cholesterol (from biosynthesis) and exogenous cholesterol (from serum). In a previous study, we used this system to establish that *C. burnetii* entry into fibroblasts occurred through lipid raft-mediated α_v_β_3_ signaling (20). In addition, with the exception of CD63, endolysosomal markers were associated with the *C. burnetii* PV regardless of the presence or absence of cholesterol, indicating that PV maturation was not cholesterol dependent. Here, we made the surprising discovery that increasing cellular cholesterol is detrimental to *C. burnetii* survival. *C. burnetii* is most sensitive to cholesterol during the early stages of infection, with increasing cholesterol levels leading to altered PV fusion, increased acidity, and bacterial degradation. Cholesterol traffics to the PV and drugs that trap cholesterol in the endolysosomal system are bactericidal, suggesting that PV cholesterol influences PV biology and *C. burnetii* pathogenesis. These data strongly support the conclusion that manipulating cholesterol in the bacterium-containing PV kills *C. burnetii*.

*C. burnetii* growth is sensitive to drugs that target cholesterol biosynthesis and uptake (19, 21). Further, Czyz et al. reported that treatment of THP-1 cells with FDA-approved drugs that alter cellular cholesterol distribution similar to U18666A also inhibit *C. burnetii* intracellular growth (19). In support of these data, they also showed decreased *C. burnetii* growth in THP-1 macrophage-like cells deficient in NPC-1, a cholesterol transporter that facilitates cholesterol export from late endosomes and lysosomes (19). We found that both plasma membrane cholesterol and the cholesterol-binding exogenous protein LDL travel to the PV. We hypothesize that cholesterol supplementation of cholesterol-free MEFs leads to increased cholesterol in the PV membrane compared to cholesterol-free MEFs. Our model system further revealed that *C. burnetii* PV size and bacterial growth are sensitive to cellular cholesterol, further supporting the hypothesis that manipulating host cell cholesterol homeostasis adversely affects *C. burnetii* infection. Importantly, our data show that rather than simply blocking bacterial growth or PV formation, increasing PV cholesterol leads to *C. burnetii* lysis. Remarkably, treatment for only 3 h with U18666A, which traps cholesterol in the PV, killed 80% of the bacteria. These data, along with data from Czyz et al. (19), suggest that *C. burnetii* is sensitive to altered cholesterol distribution within the cell, particularly accumulation of cholesterol in the endosomal trafficking pathway and the PV. With other bacteria, including *Chlamydia trachomatis*, *Staphylococcus aureus*, and *Mycobacterium* spp., the presence of cholesterol is reported to be beneficial to the bacterium, with cholesterol depletion leading to reduced bacterial growth (11, 18, 42–46). The unique sensitivity of *C. burnetii* to host cell cholesterol may reflect the distinctive intracellular niche this bacterium occupies.

Cholesterol is a key regulator of endosomal trafficking and fusion (47–49). We previously found that the endosome marker CD63 was absent in the PV lumen in cholesterol-free MEFs, suggesting that cholesterol is required for late endosomal trafficking to the PV (20). In this study, we discovered that lytic PVs containing degraded *C. burnetii*, which are found only in cells with cholesterol, were no longer fusogenic with the endosomal pathway. Most likely, bacterial degradation leads to a loss of the T4BSS effector proteins required to maintain PV fusogenicity. The *C. burnetii* T4BSS is not required for short-term intracellular survival, with a T4BSS mutant persisting for several days in a viable form (25). Thus, it is unlikely that a cholesterol-dependent loss in PV fusogenicity would lead to bacterial degradation. However, cholesterol is specifically toxic to *C. burnetii* during the initial stages of PV biogenesis and expansion, and it is possible that cholesterol plays a role in activating T4BSS secretion early during PV development. Most likely, both the timing and amount of PV cholesterol are tightly regulated by the bacteria to regulate PV-endosome fusion.

In addition to altered fusogenicity, the PVs from MEFs with cholesterol are significantly more acidic than PVs from cholesterol-free cells. Further, U18666A treatment also increased PV acidity in a vATPase-dependent manner. Importantly, blocking acidification by vATPase also rescued bacterial viability, demonstrating that increasing the PV acidity kills *C. burnetii*. This is a surprising finding, given that *C. burnetii* metabolism is activated by acid (35, 39, 40) and the PV pH has previously been reported to be approximately 4.8 (37, 38). Our studies found the PVs in cholesterol-free MEFs and HeLa cells to be slightly more alkaline at pH 5.2. The differences in measured pH between studies might be a result of different pH-sensitive reagents. We utilized the fluorescein derivative Oregon Green 488, given the improved pH sensitivity of Oregon Green 488 in acidic environments (41). A recent study on the role of lysosome-associated membrane glycoprotein (LAMP) proteins in PV maturation found the pH of the PV to be between 4.0 and 4.5 using LysoSensor Yellow/Blue DND-160 (50). In our hands, this reagent stained the bacteria and was not free in the PV lumen, prompting us to utilize the dual dextran labeling approach (41). Regardless, it is clear that the PV is more acidic in MEFs with cholesterol and HeLa cells treated with U18666A, and this increased acidity kills *C. burnetii*. However, the mechanism behind *C. burnetii* degradation is not known. Host cathepsin D and lysosomal acid phosphatases accumulate in the PV (35), and the PV is proteolytically active, presumably due to the presence of host proteases (51). It is possible that increased acidity further activates lysosomal degradative enzymes beyond the threshold the bacteria can survive. Detailed characterization of the PV proteolytic activity is needed to fully understand how *C. burnetii* survives in this environment.

Previously, a drug screen revealed that *C. burnetii* growth is sensitive to 57 FDA-approved drugs that perturb host cell cholesterol homeostasis (19, 21). We used several drugs from this screen to further validate our hypothesis that *C. burnetii* lysis was due to cholesterol-induced changes in the PV pH. Treatment with six of the eight selected drugs resulted in significant *C. burnetii* lysis which could be at least partially rescued by blocking acidification through vATPase. Importantly, these drugs were shown to have little to no effect on *C. burnetii* growth in axenic media or on host cell viability (19). While the mode of action differs between these drugs, we confirmed the results of previous studies that they altered cholesterol distribution within the cell (19). Treatment with several of these drugs appeared to increase cholesterol within the PV. Together, these findings reveal a potential vulnerability in the *C. burnetii* lifestyle which could be targeted with currently available drugs.

The cholesterol-mediated negative effect on intracellular *C. burnetii* raises intriguing questions as to how *C. burnetii* successfully colonizes cholesterol-containing cells during natural infection, given that cholesterol is an essential lipid for host cells outside the laboratory setting. Accumulating evidence suggests that *C. burnetii* possesses multiple mechanisms to manipulate host cholesterol metabolism. For example, Howe and Heinzen reported differential expression of cholesterol biosynthesis-related genes in *C. burnetii*-infected Vero cells (21). Expression profiling of *C. burnetii*-infected THP-1 cells suggests that *C. burnetii* actively upregulates expression of *apoE* and *plin2*, which are involved in cholesterol efflux and storage, respectively (26, 27). Beyond gene expression, cholesterol storage organelles called lipid droplets have been observed in and around the PVs of infected primary human alveolar macrophages (52). It is possible that *C. burnetii* targets the carefully regulated host cholesterol homeostasis, upregulating storage and efflux while also decreasing biosynthesis. In addition, we recently showed that *C. burnetii* recruits the host cell sterol-binding protein ORP1L to the PV, where it participates in membrane contact sites between the PV and endoplasmic reticulum (53). Finally, *C. burnetii* expresses two eukaryote-like sterol reductase enzymes that could modify cholesterol (54). This intriguing possibility might explain the intense filipin labeling of the PV, with a bacterium-derived β-hydroxysterol other than cholesterol dominating the PV membrane.

In summary, our data suggest that the presence of cholesterol in the PV during the initial phases of PV formation negatively affects PV formation and *C. burnetii* survival. While not absolutely required for *C. burnetii* growth, some cholesterol is needed for optimal PV development through fusion with late endosomes (20). However, too much PV membrane cholesterol leads to increased PV acidification, decreased fusion with endosomes, and eventual bacterial degradation. We propose that the amount of cholesterol in the PV membrane regulates key aspects of PV function, and *C. burnetii* must maintain a delicate balance of PV membrane cholesterol. This would explain the unique sensitivity of *C. burnetii* to drugs that target different aspects of host cholesterol metabolism: any slight shift in host cholesterol homeostasis would impact PV membrane cholesterol levels. Identifying both the bacterial and host pathways involved in this delicate balance may yield novel targets to treat or prevent *C. burnetii* pathogenesis.

## MATERIALS AND METHODS

### Bacteria and mammalian cells.

*Coxiella burnetii* Nine Mile Phase II (NMII) (clone 4, RSA439) and mCherry-expressing *C. burnetii* NMII (55) were purified from Vero cells (African green monkey kidney epithelial cells [ATCC CCL-81; American Type Culture Collection, Manassas, VA]) and stored as previously described (56). Vero cells were maintained in RPMI 1640 medium (Corning, New York, NY) containing 10% fetal bovine serum (FBS) (Atlanta Biologicals, Norcross, GA) at 37°C and 5% CO_2_. DHCR24^−/−^ mouse embryonic fibroblasts (MEFs) were cultured in fibroblast media supplemented with serum-free growth kit (ATCC) and cholesterol (Synthechol; Sigma-Aldrich, St. Louis, MO) as previously described (20). The multiplicity of infection (MOI) was optimized for each bacterial stock, cell type, and infection condition for a final infection of ca. one internalized bacterium/cell at 37°C and 5% CO_2_.

### PV measurements.

A total of 5 × 10^4^ MEFs were plated onto ibidi-treated channel µslide VI^0.4^ (3 × 10^3^ cells per channel; ibidi USA Inc., Verona, WI) and allowed to adhere overnight. After the MEFs were infected with *C. burnetii* for 1 h, they were washed with phosphate-buffered saline (PBS) to remove extracellular bacteria and incubated in media containing the indicated cholesterol concentrations. At different time points postinfection, cells were fixed with 2.5% paraformaldehyde (PFA) on ice for 15 min and then permeabilized/blocked for 15 min with 0.1% saponin and 1% bovine serum albumin (BSA) in PBS. The cells were incubated with rat anti-LAMP1 (catalog no. 553792; BD Biosciences, San Jose, CA) and rabbit anti-*C. burnetii* primary antibodies in saponin-BSA-PBS for 1 h, followed by Alexa Fluor secondary antibodies (Invitrogen) for 1 h. Following washing with PBS, ProLong Gold with 4′,6′-diamidino-2-phenylindole (DAPI) (Invitrogen) was added, and the cells on the slides were visualized on a Leica inverted DMI6000B microscope (63× oil immersion objective). Images were captured and processed identically, and a cross-sectional area through the middle of the PV was measured using ImageJ software. Approximately 20 PVs were measured per condition for each of three independent experiments.

### *C. burnetii* growth in MEFs.

MEFs were plated at 1 × 10^5^ cells/well in a six-well plate under different cholesterol conditions and allowed to adhere overnight. After the MEFs were infected with *C. burnetii* for 1 h in 500 µl medium, the wells were washed with PBS to remove extracellular bacteria and then gently scraped into 3 ml of medium. For the day 0 sample, 1 ml of the cell suspension was centrifuged at 20,000 × *g* for 10 min, and the pellet was frozen at −20°C. The remaining cells were left in the six-well plate in medium supplemented with cholesterol. The medium was changed daily to ensure constant cholesterol concentrations. At 6 days postinfection, the cells were harvested by scraping the cells into the growth medium and centrifuging at 20,000 × *g* for 10 min. Bacterial DNA was extracted from the pellets using the UltraClean microbial DNA isolation kit (Mo Bio Laboratories, Carlsbad, CA) according to the manufacturer’s instructions. Quantitative PCR for genome equivalents was performed using a primer set specific for *dotA* (30) and Luminaris Color HiGreen quantitative PCR (qPCR) master mix (Thermo Scientific) with an Applied Biosystems 7500 real-time PCR cycler. Each experiment was done in duplicate.

### Quantitation of lytic PVs containing lysed *C. burnetii.*

DHCR24^−/−^ MEFs were plated under different cholesterol conditions at 5 × 10^4^ cells per well of a six-well plate and infected with mCherry-expressing *C*. *burnetii* (mCherry-*C. burnetii*) for 1 h as described above. Approximately 24 h later, the cells were scraped into fresh medium, resuspended to 1 × 10^5^ cells/ml, and plated onto ibidi-treated channel µslide VI^0.4^ (3 × 10^3^ cells per channel). The medium was changed daily, and cells were examined live every 24 h on a Leica inverted DMI6000B microscope with a 63× oil immersion objective. PVs with visible mCherry fluorescence in the PV lumen were scored as “lytic PVs” with 50 PVs scored for each condition for three individual experiments.

HeLa cells (5 × 10^4^) were infected with mCherry-*C. burnetii* in 6-well plates for 1 h. At 2 days postinfection, the cells were trypsinized and resuspended to 1 × 10^5^ cells/ml, and plated onto ibidi-treated channel µslide VI^0.4^ (3 × 10^3^ cells per channel; Ibidi). At 3 days postinfection, dimethyl sulfoxide (DMSO) control, U18666A (1 or 5 µM), or the indicated FDA-approved drugs (see Table S1 in the supplemental material; obtained from Sigma and used at a final concentration of 20 µM) with or without vATPase inhibitor bafilomycin A1 (100 nM) were added to the cells and incubated for the time indicated prior to counting lytic PVs as described above. At least 50 PVs were scored for each condition for three individual experiments.

### *C. burnetii* viability by fluorescent infectious focus-forming unit (FFU) assay.

To test viability of *C. burnetii* in MEFs under different cholesterol conditions, 1 × 10^4^ cells/well were infected with *C. burnetii* for 1 h in a 48-well plate, washed extensively with PBS, and incubated with media containing different cholesterol concentrations. At the indicated time points, cells were incubated for 5 min with sterile water, pipetted up and down to lyse cells, and diluted 1:5 in RPMI 1640 with 2% FBS (2% FBS-RPMI). Serial dilutions were added to confluent monolayers of Vero cells in a 24-well plate and incubated for 5 days. Plates were fixed with methanol and stained with rabbit anti-*C. burnetii* antibody and DAPI to confirm monolayer integrity. Four fields per well were captured on an Evos automated microscope (Thermo Fisher) with a 4X objective, and fluorescent focus units were quantitated using ImageJ. Each experiment was done in duplicate.

To determine bacterial viability in drug-treated cells, HeLa cells were plated at 5 × 10^4^ cells/well in a six-well plate and infected with mCherry-*C. burnetii*. At 2 days postinfection, the cells were trypsinized and replated in 24-well plates at 5 × 10^4^ cells/well. Approximately 16 h later, the cells were treated with DMSO or drug with or without the vATPase inhibitor bafilomycin A1 (100 nM) for the time indicated, at which point the medium was aspirated from the 24-well plate and the cells were lysed by incubation in sterile water for 5 min. After the cells were pipetted up and down, the released bacteria were diluted 1:5 in 2% FBS-RPMI and plated in 10-fold serial dilutions onto confluent Vero cell monolayers in a 96-well ibidi-treated µplate (ibidi). The plate was fixed with 2.5% PFA 5 days later and stained with DAPI, and the number of fluorescent foci was determined as described above. Each experiment was done in duplicate.

### Microscopy for cholesterol trafficking.

To monitor trafficking of plasma membrane cholesterol, fluorescent cholesterol (TopFluor cholesterol; Avanti Polar Lipids) was resuspended at 20 mg/ml in ethanol. Twenty microliters of this solution was added to 1 ml of 10% methyl-beta-cyclodextrin (Sigma) in serum-free RPMI 1640 medium. The solution was sonicated in a water bath sonicator (Avanti) for 30 s, and insoluble material was pelleted by spinning for 2 min at 20,000 × *g*. MEFs with cholesterol were infected with mCherry-*C. burnetii* and plated onto an ibidi µslide as described above. At 3 days postinfection, fluorescent cholesterol (final concentration of 30 µg/ml) was added to the cells for 24 h. Live-cell images were taken with a modified PerkinElmer UltraView spinning disk confocal connected to a Nikon Eclipse Ti-E inverted microscope with a 63× oil immersion objective.

For trafficking of BODIPY-LDL, MEFs were infected and plated onto an ibidi µslide as described above. The cells were incubated for 5 min on ice with 25 µg/ml BODIPY-LDL (Invitrogen), washed twice with medium, and visualized after 4 h of incubation at 37°C.

To visualize endogenous free sterols after drug treatment, infected cells were plated onto ibidi µslides and treated as described above. The cells were fixed with 2.5% PFA on ice for 15 min and incubated with 1:100 filipin (Cayman Chemicals, Ann Arbor, MI) in PBS with 1% BSA for 1 h. After the cells were washed with PBS three times, they were incubated with rat anti-LAMP1 for MEFs (catalog no. 553792; BD Biosciences, San Jose, CA) or mouse anti-CD63 for HeLa cells for 1 h, followed by three washes in PBS and a 1 h incubation with Alexa Fluor 488 anti-mouse secondary antibody. After the cells were washed three times with PBS, ProLong Gold was added to the wells, and samples were visualized on a Leica inverted DMI6000B microscope (63× oil immersion objective). Images were captured under identical capture settings and processed identically using ImageJ.

### *C. burnetii* growth in cell-free media.

To complex cholesterol to BSA, 500 µg of cholesterol (10 mg/ml chloroform stock; Avanti) was dried down in a glass tube under a nitrogen stream. The lipid film was resuspended into 2.5 ml of 5% fatty acid-free BSA using a water bath sonicator (Avanti). The resulting 200 µg/ml stock was sterile filtered and added to a final concentration of 5 µg/ml in ACCM-2 (40). *C. burnetii* bacteria were diluted to approximately 1 × 10^5^ genomes/ml in ACCM-2 with BSA or BSA-cholesterol, and 7 ml was transferred to a T25 flask and incubated as previously described (40). Every 24 h, 50 µl was removed and added to a tube with 150 µl PBS and a half volume of 0.1-mm zirconia-silica beads (BioSpec Products, Bartlesville, OK). Bacteria were lysed by bead beating in a FastPrep FP120 (Thermo Scientific) and analyzed by qPCR as previously described (20). Each experiment was done in duplicate.

To test bacterial sensitivity to U18666A, ACCM-2 was inoculated at approximately 1 × 10^5^ bacteria/ml with mCherry-*C. burnetii* and grown for 5 days as previously described (40). Bacteria (500 µl) were treated for 6 h with DMSO or U18666A in 24-well plates under normal *C. burnetii* culture conditions. The bacteria were diluted 1:10 in 2% FBS-RPMI prior to the FFU assay in 96-well ibidi-treated µplates as described above.

### Dextran trafficking.

Cells were infected with mCherry-*C. burnetii* in six-well plates and replated onto ibidi slides at 2 days postinfection as described above. PVs were selected and marked in Elements software on the spinning disk confocal microscope in a live-cell environmental chamber. Individual ibidi channels were pulsed with 1 mg/ml Alexa Fluor 488-dextran (molecular weight [MW] of 10,000) for 10 min in medium, followed by four washes with medium to remove uninternalized dextran and finally replaced with either basal medium or basal medium with cholesterol (5 µg/ml). The PVs were then focused, and confocal images through the entire PV were obtained every 6.33 min for 38 min. The fluorescence intensity of dextran inside the PV was calculated using the average intensity multiplied by the PV volume using ImageJ.

### PV pH measurements.

The pH measurement was performed as previously described with slight modifications (57). Briefly, MEFs were infected with mCherry-*C. burnetii* in six-well plates, incubated with and without cholesterol, and replated onto ibidi slides at 2 days postinfection as described above. For measurement of PV pH in U18666A-treated cells, HeLa cells were infected with mCherry-*C. burnetii* in six-well plates and replated onto ibidi plates at 2 days postinfection. Under both conditions, at 3 days postinfection, cells were incubated with pH-sensitive Oregon Green 488 dextran (MW, 10,000; Invitrogen) and pH-stable Alexa Fluor 647 dextran (MW, 10,000; Invitrogen) for 4 h at a concentration of 0.5 mg/ml. MEFs were imaged directly with a 63× oil immersion objective under identical capture settings.

To measure the time-dependent change in PV pH, individual PVs were selected and imaged, and then treated with 5 µM U18666A or DMSO as a vehicle control. Starting from 15 min after the treatment, cells were then imaged every 15 min for the next 2 h. The PV fluorescence intensity was measured using ImageJ, and the Oregon Green 488/Alexa Fluor 647 ratio was calculated. To generate a standard curve for MEFs and HeLa cells, the respective infected cells were incubated with the ionophores nigericin (10 µM) and monensin (10 µM) for 5 min at room temperature, followed by buffers with different pHs (pH 4.0 to 7.0) before imaging. At least 20 PVs were imaged at each pH for every experiment, and the ratio of fluorescence intensity at 488/647 nm were plotted against the pH of the respective buffer to obtain a sigmoidal standard curve.

### Data analyses.

Image processing and analysis were done with ImageJ software (W. S. Rasband, National Institutes of Health, Bethesda, MD) (58). Statistical analyses were performed using unpaired two-tailed *t* test, ordinary one-way or two-way analysis of variance (ANOVA) with Tukey’s or Dunnett’s multiple-comparison test in Prism (GraphPad Software, Inc., La Jolla, CA).
