# Supplementary figures and images for: Elevated Cholesterol in the Coxiella burnetii Intracellular Niche Is Bacteriolytic
Source: mBio. 2017 Feb 28;8(1):e02313-16. doi: 10.1128/mBio.02313-16 (PMC5347348; doi:10.1128/mBio.02313-16)

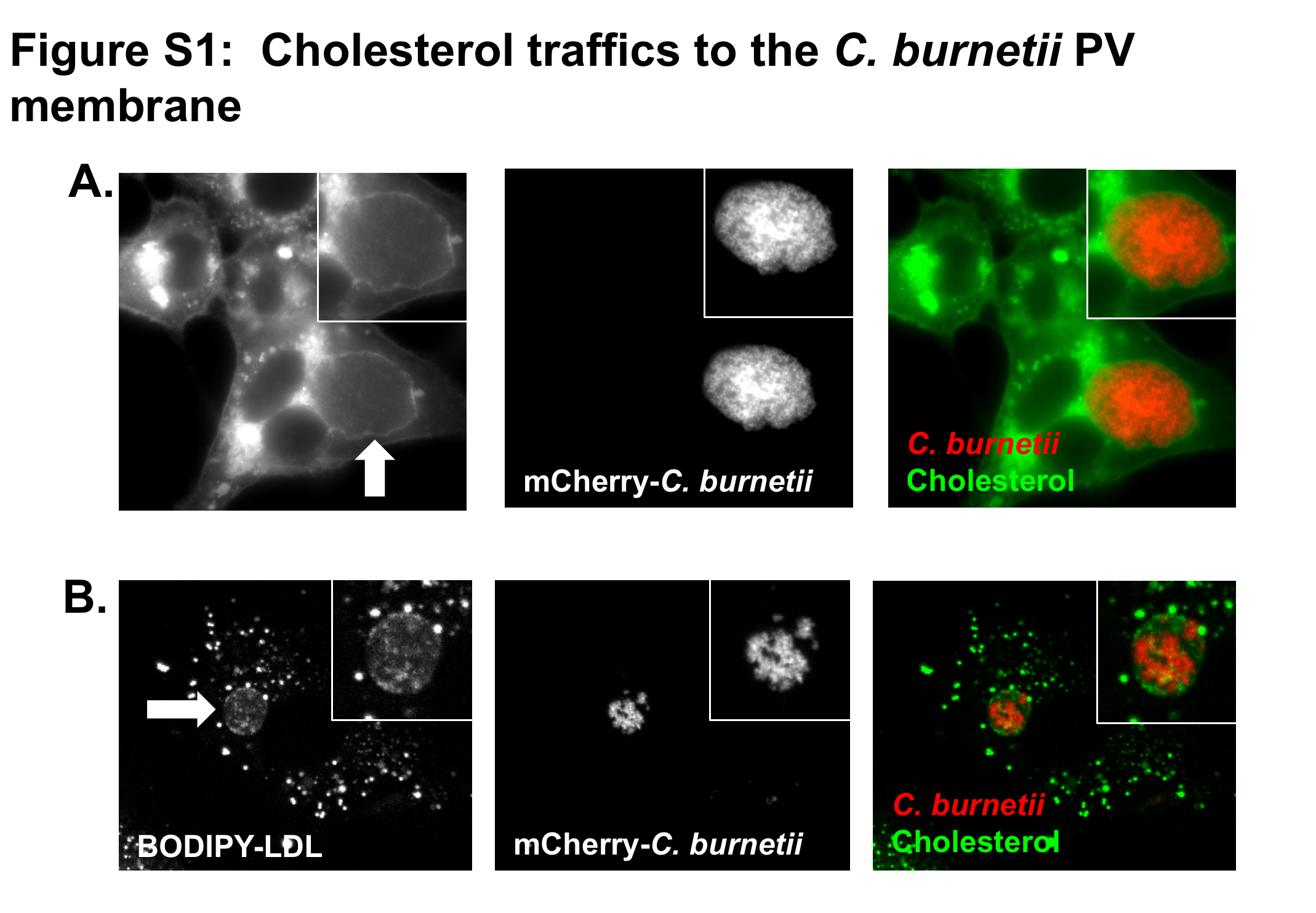

Supplement: FIG S1 [file mbo001173208sf1.tif]

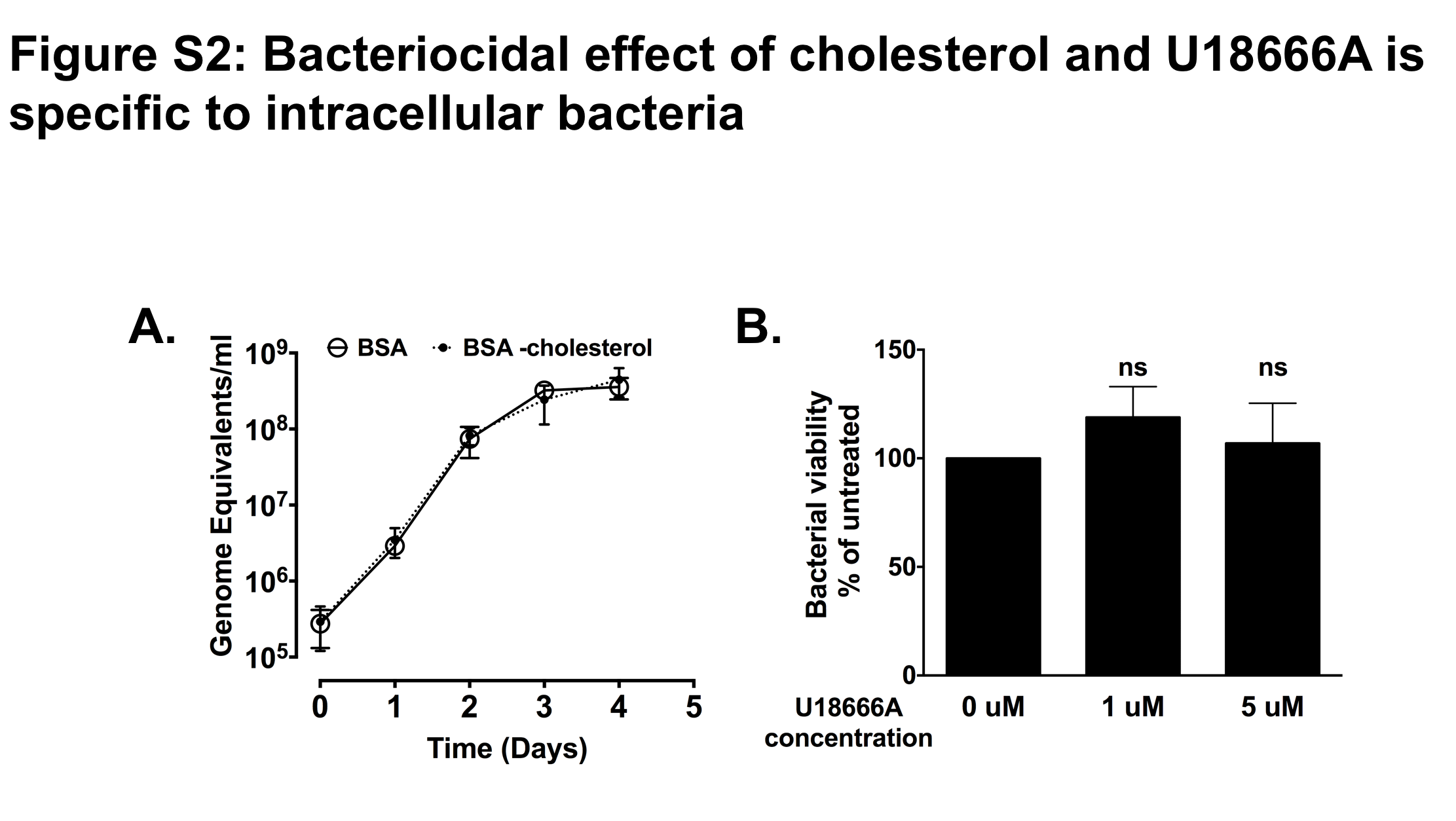

Supplement: FIG S2 [file mbo001173208sf2.tif]

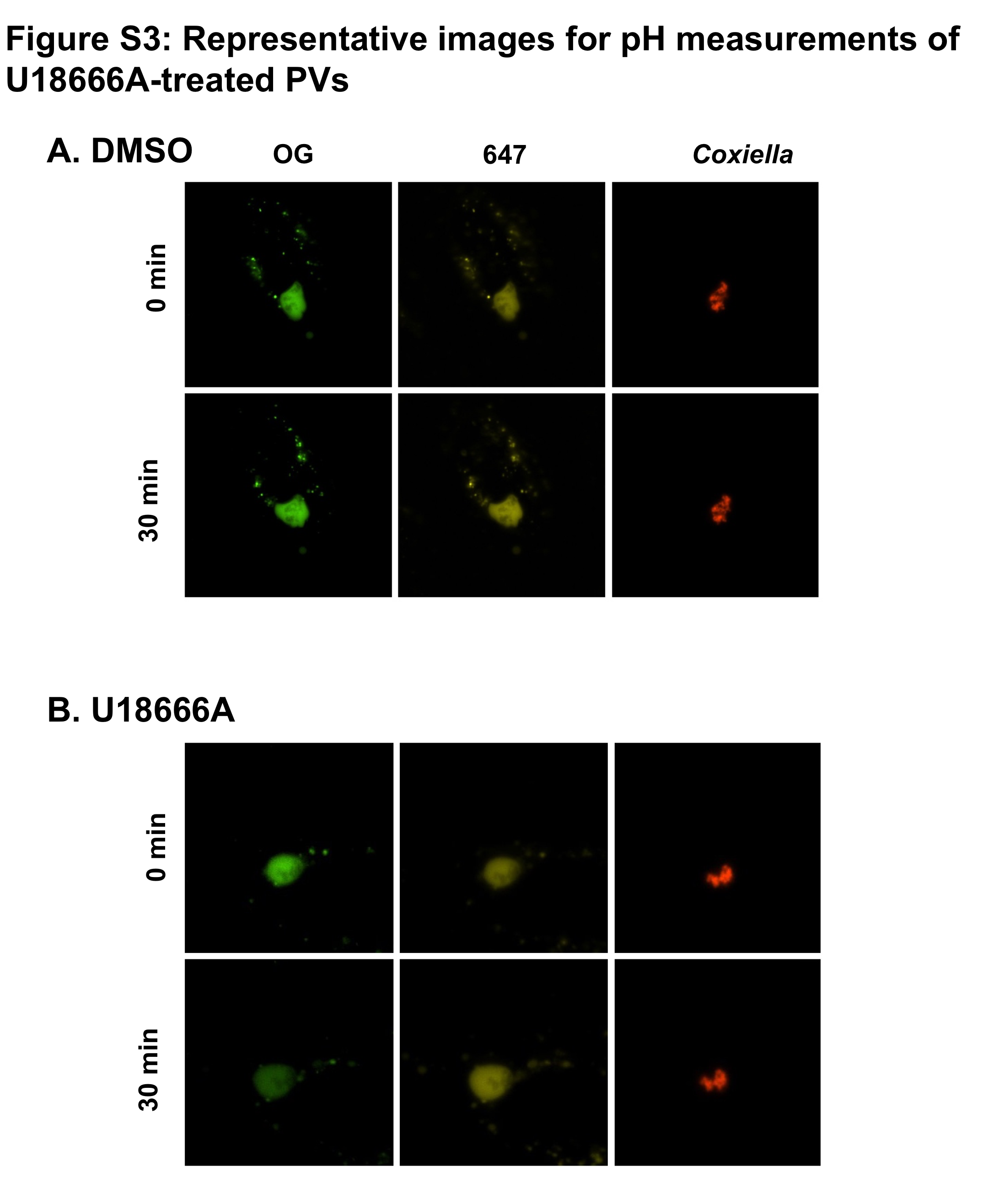

Supplement: FIG S3 [file mbo001173208sf3.tif]

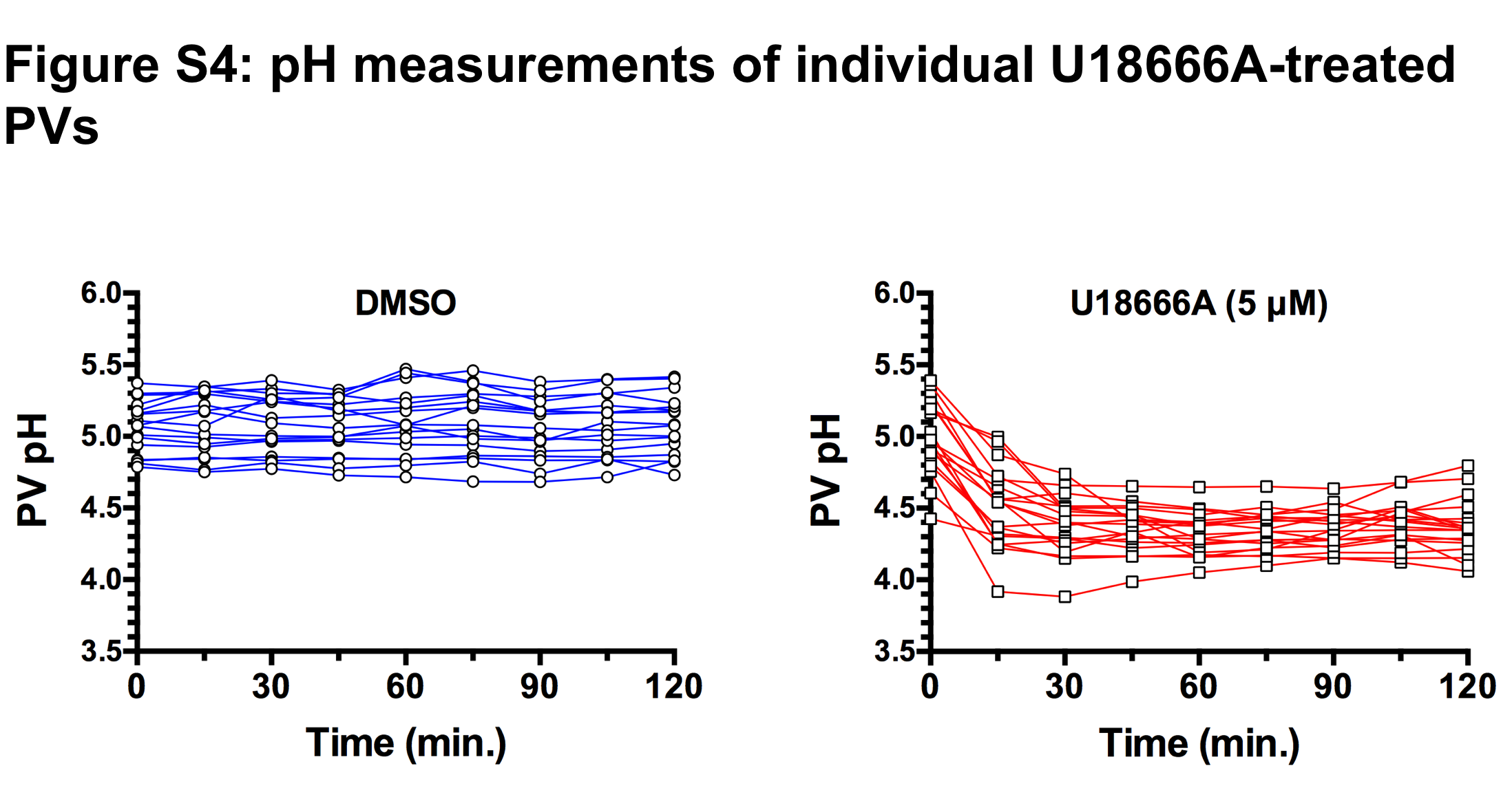

Supplement: FIG S4 [file mbo001173208sf4.tif]

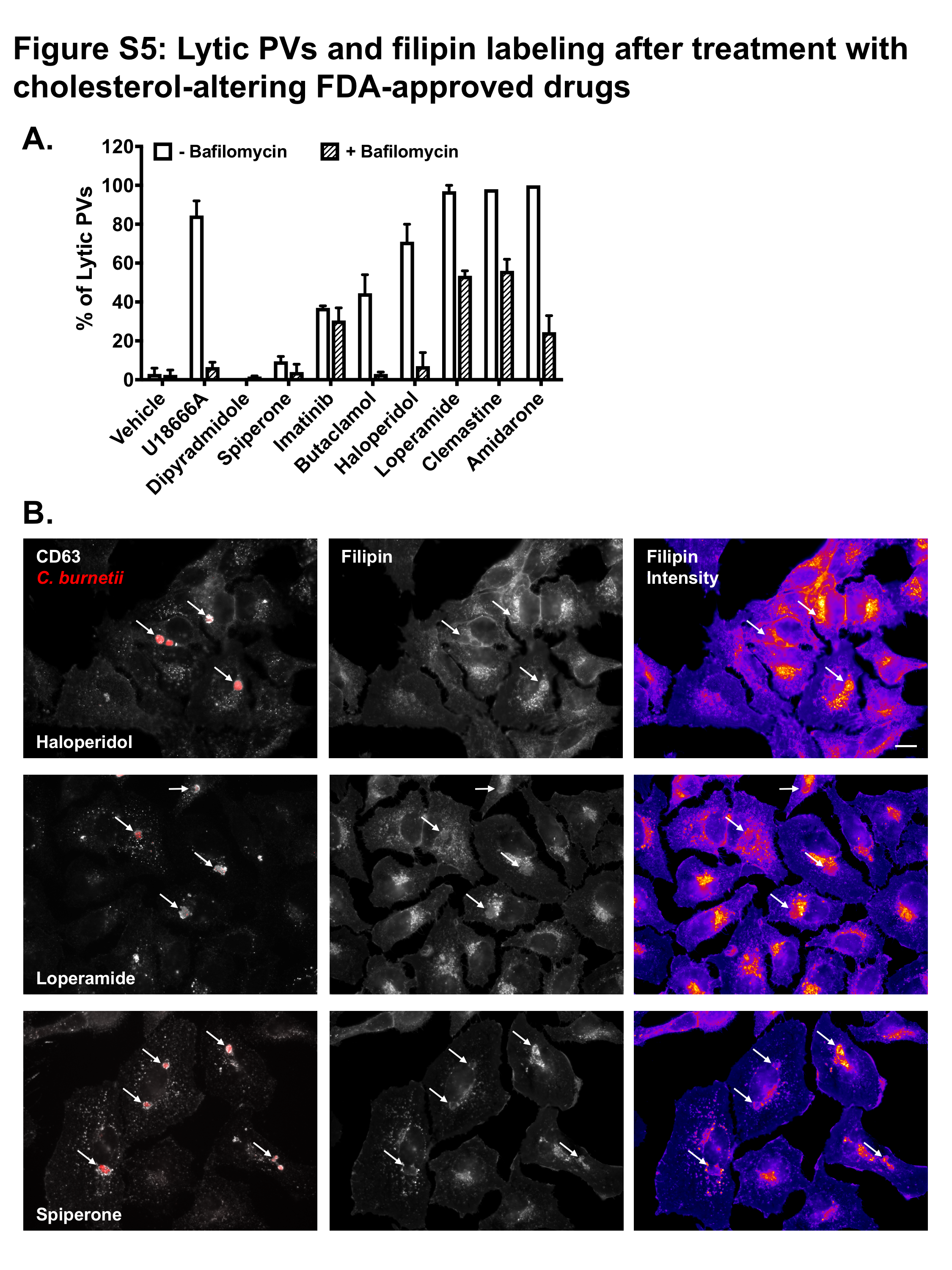

Supplement: FIG S5 [file mbo001173208sf5.tif]
