# Supplementary material for: Elevated Cholesterol in the Coxiella burnetii Intracellular Niche Is Bacteriolytic
Source: mBio. 2017 Feb 28;8(1):e02313-16. doi: 10.1128/mBio.02313-16 (PMC5347348; doi:10.1128/mBio.02313-16)
Supplement: TABLE S1 [file mbo001173208st6.docx]

**Supplemental Table S1: FDA approved cholesterol altering drugs.**

| **Drug** | **Therapeutic Class** | **Putative pathway targeted** | **Effect on *C. burnetii* intracellular growth in THP-1 cells over 5 days relative to vehicle, (10uM) (19)** | **Effect on growth in axenic medium, relative to vehicle (10uM) (19)** | **% of Lytic PVs (this study)** | **Bafilomycin treatment**  **(% of lytic PVs) (this study)** | **Increased filipin in PV lumen (this study)** | **Increased filipin intensity in endolysosomal vesicles (this study)** |
| --- | --- | --- | --- | --- | --- | --- | --- | --- |
| Vehicle |  |  | 1 | 1 | 3 | 2.5 | No | No |
| U18666A | Positive Control |  |  |  | 84.5 | 6.5 | + | + |
| Dipyramidole | Antithrombotic | PDE ^a^ | 0.9 | 0.96 | 0 | 1.5 | ND | ND |
| Spiperone.HCl | Antipsychotic | GPCRs ^b^ /Ca | 0.8 | 0.89 | 15.5 | 4 | +/- | - |
| Imatinib | Anitneoplastic |  | 0.61 | 1.05 | 37 | 30.5 | ND | ND |
| Butaclamol. HCl | Antipsychotic | GPCRs ^b^ /Ca | 0.84 | 0.99 | 44.5 | 3 | ND | ND |
| Haloperidol | Antipsychotic | GPCRs ^b^ /Ca | 0.87 | 0.95 | 71 | 7 | - | + |
| Loperamide.HCl | Antidiarrheal | GPCRs ^b^ /Ca | 0.26 | 0.91 | 97 | 53.5 | + | + |
| Clemastine | Antihistamine | GPCRs ^b^ | 0.44 | 1.11 | 98 | 56 | - | + |
| Amiodarone.HCl | Antiarrhythmic | IonC ^c^ | 0.61 | 0.9 | 100 | 24.5 | + | - |

^a^ Phosphodiesterase ^b^ G-Protein coupled receptors ^c^ Ion Channel ND not done
